# Anti-apoptotic Effects of PCP4/PEP19 in Human Breast Cancer Cell Lines: A Novel Oncotarget

**DOI:** 10.18632/oncotarget.2161

**Published:** 2014-07-08

**Authors:** Taiji Hamada, Masakazu Souda, Takuya Yoshimura, Shoko Sasaguri, Kazuhito Hatanaka, Takashi Tasaki, Takako Yoshioka, Yasuyo Ohi, Sohsuke Yamada, Masato Tsutsui, Yoshihisa Umekita, Akihide Tanimoto

**Affiliations:** ^1^ Department of Molecular and Cellular Pathology, Kagoshima University Graduate School of Medical and Dental Sciences, Kagoshima, Japan; ^2^ Department of Oral and Maxillofacial Surgery, Kagoshima University Graduate School of Medical and Dental Sciences, Kagoshima, Japan; ^3^ Department of Pathology, Sagara Hospital, Social Medical Corporation Hakuaikai, Kagoshima, Japan; ^4^ Department of Pathology and Cell Biology, School of Medicine, University of Occupational and Environmental Health, Kitakyushu, Japan; ^5^ Department of Pharmacology, Faculty of Medicine, University of the Ryukyus, Okinawa, Japan; ^6^ Division of Organ Pathology, Faculty of Medicine, Tottori University, Yonago, Japan

**Keywords:** PCP4/PEP19, breast cancer, apoptosis, CaMKK, Akt

## Abstract

The PCP4/PEP19 is a calmodulin-binding anti-apoptotic peptide in neural cells but its potential role in human cancer has largely been unknown. We investigated the expression of PCP4/PEP19 in human breast cancer cell lines MCF-7, SK-BR-3, and MDA-MB-231 cells, and found that estrogen receptor (ER)-positive MCF-7 and ER-negative SK-BR-3 cells expressed PCP4/PEP19. In the MCF-7 cells, cell proliferation was estrogen-dependent, and PCP4/PEP19 expression was induced by estrogen. In both cell lines, PCP4/PEP19 knockdown induced apoptosis and slightly decreased Akt phosphorylation. Knockdown of calcium/calmodulin-dependent protein kinase kinase 1 (CaMKK1), resulting in decreased phospho-Akt^Thr308^, enhanced apoptosis in SK-BR-3 but not in MCF-7 cells. CaMKK2 knockdown moderately decreased phospho-Akt^Thr308^ and increased apoptosis in MCF-7 cells but not in SK-BR-3 cells. These data indicated that PCP4/PEP19 regulates apoptosis but exact mechanism is still unknown. PCP4/PEP19 can therefore potentially serve as independent oncotarget for therapy of PCP4/PEP19-positive breast cancers irrespective of ER expression.

## INTRODUCTION

The Purkinje cell protein 4 (PCP4), which is also known as peptide 19 (PEP19) was first identified in rat cerebellar extracts as a developmentally regulated polypeptide of 7.6 kDa that has regions homologous to the calcium binding β-chain of the S100 protein [1]. PCP4/PEP19 is a calmodulin (CaM)-binding protein and regulates CaM-dependent signaling [2-4]. Immunocytochemical evidence showed PCP4/PEP19 to be present in the Purkinje cells and stellate neurons of rat cerebellum [5]. Expression of PCP4/PEP19 modulates calcium/CaM-dependent protein kinase (CaMK) activity through binding to calmodulin [6, 7]. Consequently, PCP4/PEP19 might affect a variety of cellular functions involved in the control of various neuronal processes, including apoptosis (reviewed in ref. 8). Indeed, PCP4/PEP19 has been reported to have anti-apoptotic effects on rat chromaffin PC12 and HEK293T cells [9-11].

Although PCP4/PEP19 has been reported to be predominantly expressed in the central nervous system, it is also known to be expressed in other human organs, including prostate, kidney, and uterus. In the uterus, especially, benign leiomyoma expressed PCP4/PEP19 to a greater degree compared with non-neoplastic myometrium [12]. Recent studies have demonstrated PCP4/PEP19 expression in the normal and neoplastic human adrenocortical tissues, acting as a regulator of aldosterone production [13, 14]. However, an association between PCP4/PEP19 expression and cancer cell apoptosis has not been fully investigated yet and is largely disregarded in current discussion, even though our previous study showed that PCP4/PEP19 expression in the mammary gland tissue was significantly increased during rat carcinogenesis stimulated by 7, 12-dimethylbenz [a] anthracene (DMBA) exposure [15]. The expression of PCP4/PEP19 was significantly induced in the terminal end buds where mammary gland carcinoma develops, and the expression levels increased as cancer progressed from ductal carcinoma *in situ* to invasive ductal carcinoma. Given that estrogen increased the expression of PCP4/PEP19 and the fact that development of DMBA-induced rat mammary gland carcinoma are highly estrogen-dependent [16, 17], PCP4/PEP19 expression may be deduced to be under the regulation of estrogen during mammary carcinogenesis. More specifically, we speculated that the expression of PCP4/PEP19 would be up-regulated by estrogen and mediate anti-apoptotic functions in human breast cancer cells. We investigated the expression of PCP4/PEP19 in the human breast cancer cell lines, MCF-7, SK-BR-3, and MDA-MD-231, and found that it was expressed in both estrogen receptor (ER)-positive MCF-7 and ER-negative SK-BR-3 cells. We further unveiled PCP4/PEP19 function as an anti-apoptotic factor potentially acting through Akt signaling pathways involving different isoforms of the calcium/CaM-dependent protein kinase kinase (CaMKK). To our knowledge, we are the first to demonstrate that PCP4/PEP19 actively prevents apoptosis in human breast cancer cells, suggesting that PCP4/PEP19 can potentially serve as a novel drug target to enhance apoptotic cell death irrespective of the status of ER expression.

## RESULTS

### The effects of estrogen on cell proliferation and PCP4/PEP19 expression in human breast cancer cell lines

**Figure 1:**
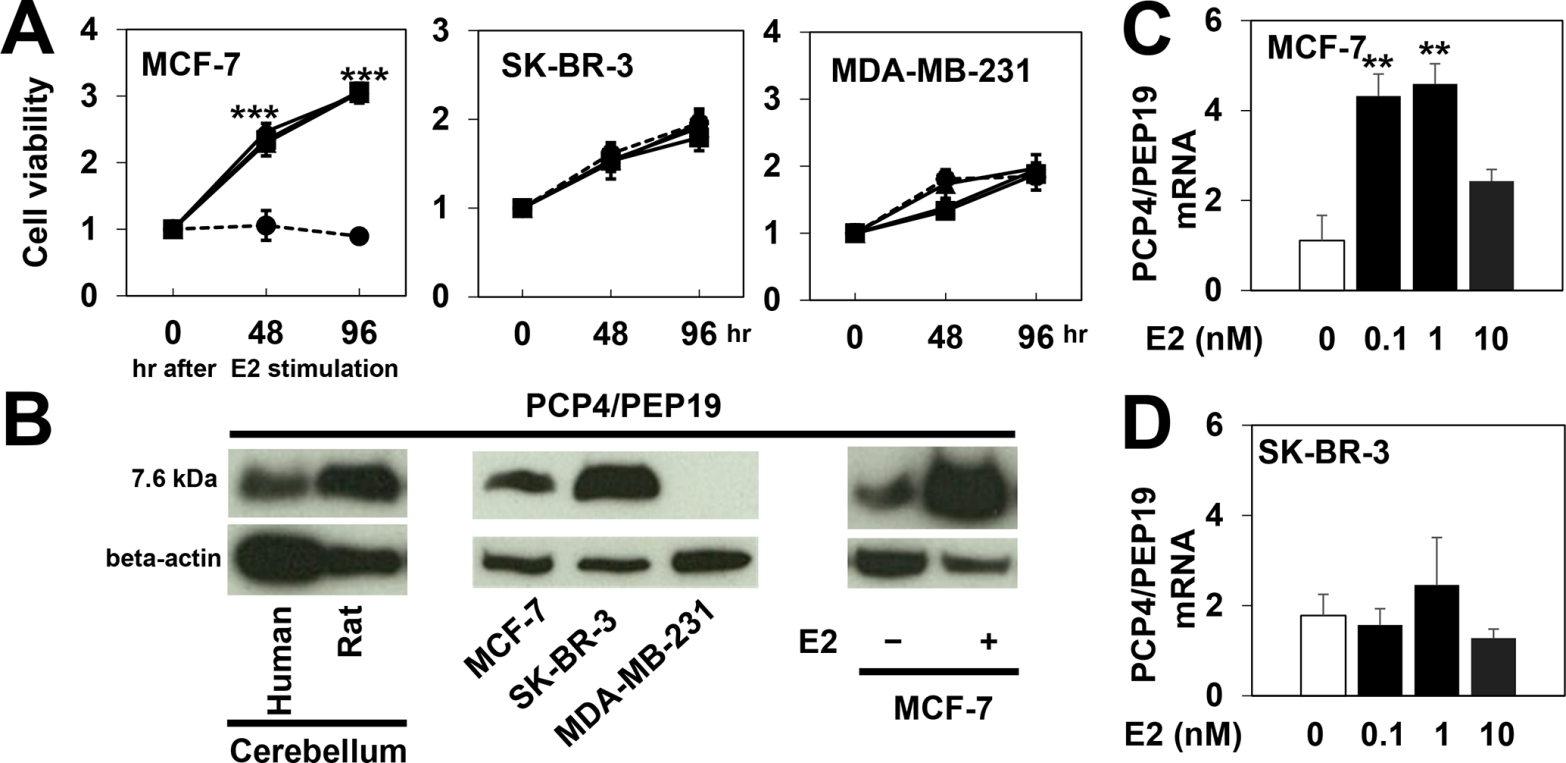
PCP4/PEP19 expression and cell proliferation of human breast cancer cell lines **A**) Cell number was monitored in the presence and absence of E2 in MCF-7, SK-BR-3, and MDA-MB-231 cells for 96 hr. The values were presented as fold increase over those in 0 hr (n=6). In MCF-7 cells, no proliferation was observed in the absence of E2 (dashed line). E2 significantly stimulated MCF-7 cells to proliferate (triangle, 0.1nM; square, 1nM; circle, 10nM E2). SK-BR-3 and MDA-MB-231 cells proliferated even without E2. **B**) Western blot analysis of PCP4/PEP19, shown to be constitutively expressed in human and rat cerebellum (left). MCF-7 and SK-BR-3 but not MDA-MB-231 cells expressed PCP4/PEP19 (middle) and ER-positive MCF-7 cells were stimulated to express PCP4/PEP19 with 1 nM E2 (right). **C**), **D**) PCP4/PEP19 mRNA expression was induced by E2 treatment for 96 hr in MCF-7 cells but not in ER-negative SK-BR-3 cells (n=6). **, p<0.01 and ***, p<0.001 versus 0 nM E2.

Cells from three human breast cancer cell lines, MCF-7, SK-BR-3 and MDA-MD-231, were cultured and incubated with 0, 0.1, 1 and 10 nM 17-beta estradiol (E2) in the medium supplemented with 10% charcoal-stripped FBS and cell proliferation was monitored by the WST-8 assay for 96 hr. The MCF-7 cells, which express ER, did not proliferate without E2 supplementation in the medium (Fig. 1A, dashed line in left panel) and were stimulated to proliferate with 0.1 and 1 nM E2 treatments. In contrast, ER-deficient SK-BR-3 and MDA-MB231 cells proliferated even with no E2 stimulation (Fig. 1A, middle and right panels). The expression of PCP4/PEP19, constitutively expressed in human and rat cerebellum (Fig. 1B, left panel), was detected in MCF-7 and SK-BR-3 cells but not in MDA-MD-231 cells (Fig. 1B, middle panel), and the protein and mRNA expression levels were markedly induced by E2 treatment in MCF-7 cells but not in SK-BR-3 cells (Fig. 1B, right panel, Figs. 1C and 1D).

Therefore, MCF-7 and SK-BR-3 cells were used for further studies to investigate the functions of PCP4/PEP19. Furthermore, for experiments using MCF-7 cells, media containing 1 nM E2 were used to study the effects of PCP4/PEP19 on cellular proliferation.

### Effects of PEP19/PCP4 knockdown on cancer cell proliferation

**Figure 2:**
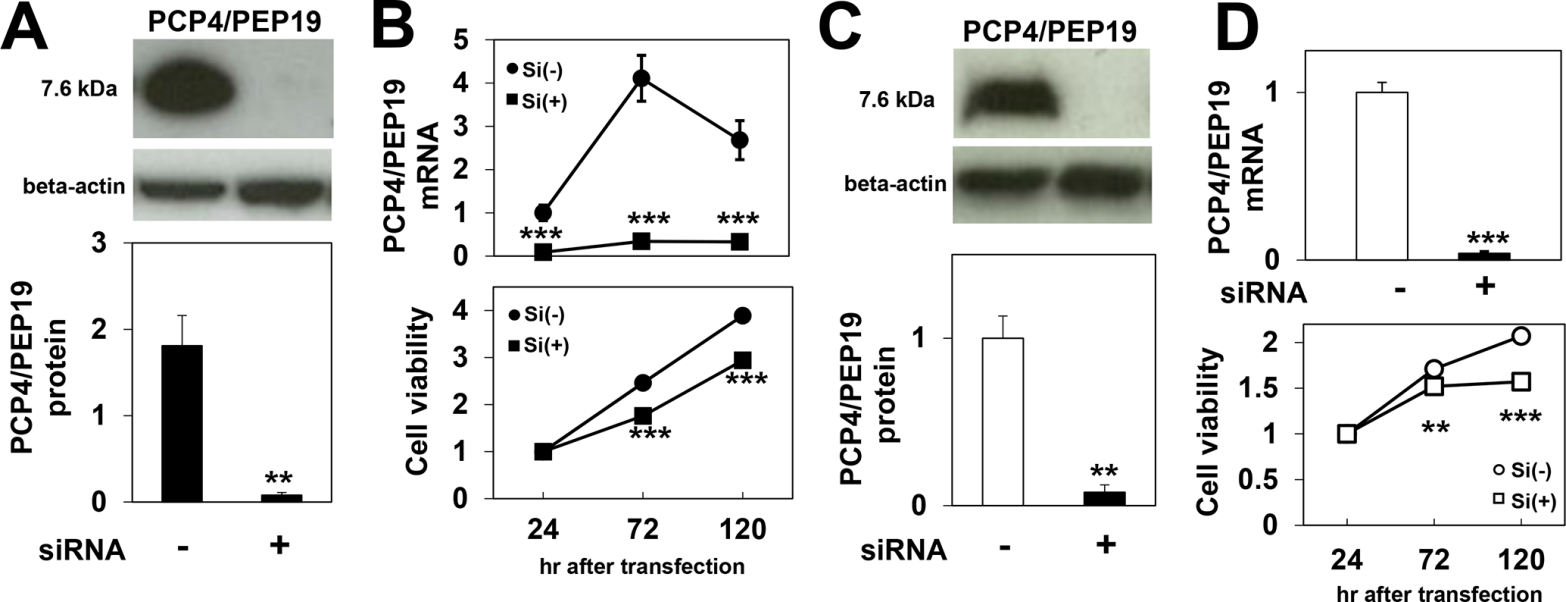
Effects of PCP4/PEP19 knockdown on cell proliferation **A**), **B**) PCP4/PEP19 expression in MCF-7 cells was reduced by siRNA both at the protein (n=3) and mRNA levels (n=6) 72 hr after siRNA transfection (A). Decreases in PCP4/PEP19 mRNA expression and cell numbers were monitored until 120 hr after siRNA transfection (B) (n=6). **C**), **D**) Reduced expression of PCP4/PEP19 (72 hr after siRNA transfection) and cell proliferation were also observed in SK-BR-3 cells by siRNA treatment. **, p<0.01 and ***, p<0.001 versus negative control siRNA. Si(-), negative control siRNA; Si(+), PCP4/PEP19 siRNA

When ER-positive MCF-7 cells were stimulated with 1 nM E2, the expression levels of PCP4/PEP19 proteins were markedly increased, and, in those targeted with PEP19/PCP4-specific siRNA, the protein expression was found to have decreased (Fig. 2A). The PCP4/PEP19 mRNA expression levels also significantly increased by 1 nM E2 treatment and were down-regulated by siRNA treatment (Fig. 2B, upper panel). The WST-8 assay showed that PCP4/PEP19 mRNA knockdown reduced the viable cell counts in the presence of E2 in the culture medium (Fig. 2B, lower panel). In SK-BR-3 cells, the protein and mRNA expression levels of PCP4/PEP19 were also down-regulated by PCP4/PEP19 siRNA (Figs. 2C and 2D), decreasing the viable cell count in this strain as well (Fig. 2D, lower panel).

### Enhanced apoptosis by PCP4/PEP19 knockdown

**Figure 3:**
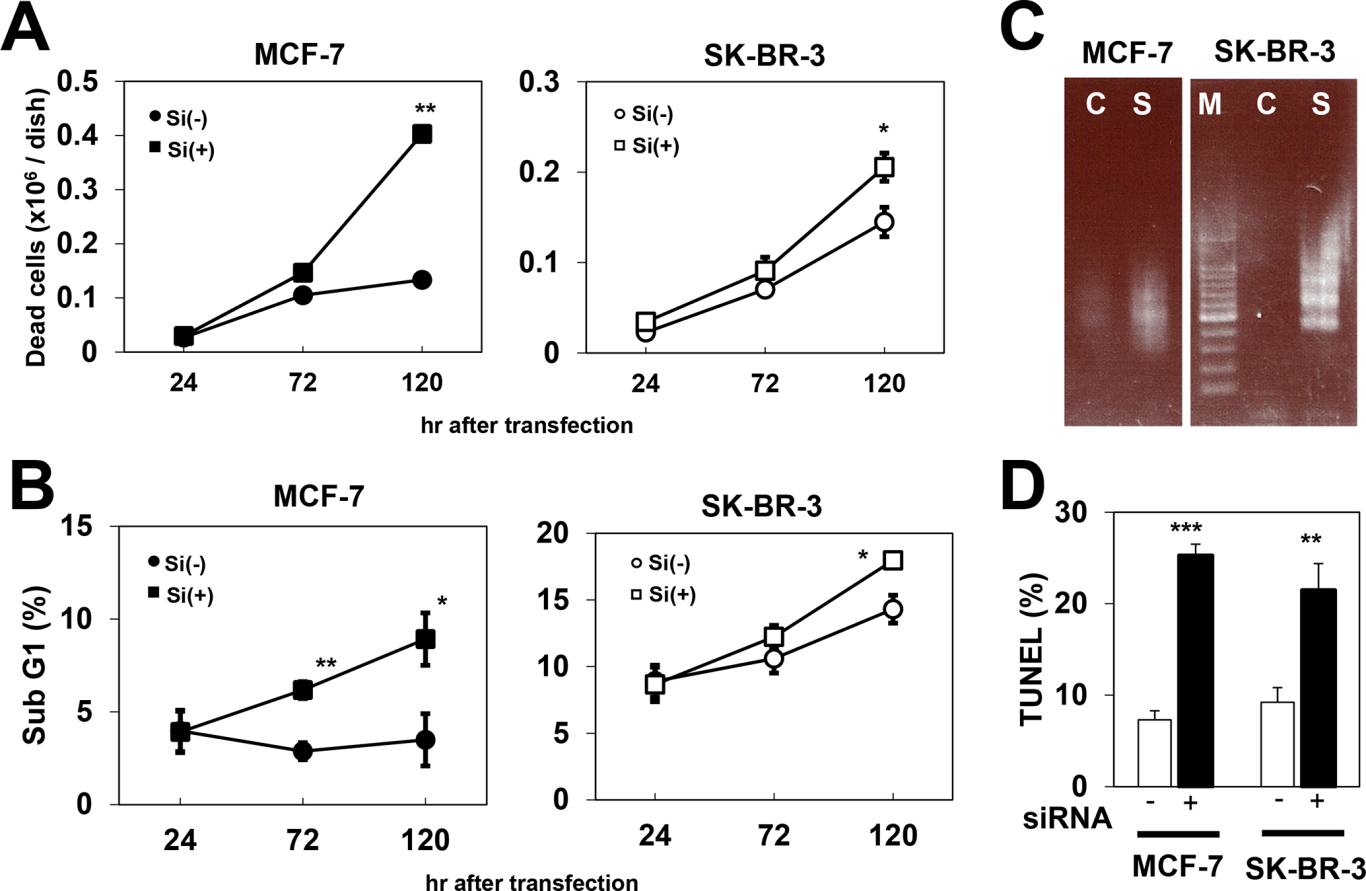
Increased cell death due to apoptosis by PCP4/PEP19 knockdown **A**) Trypan blue exclusion test showed increased dead cell numbers after PCP4/PEP19 knockdown (n=6). **B**) Flow cytometry revealed that sub G1 fraction was also increased in MCF-7 and SK-BR-3 cells by PCP4/PEP19 siRNA (n=3). **C**) Ladder formation of genomic DNA obtained from PCP4/PEP19 knockdown cells 168 hr after siRNA transfection. **D**) TUNEL assay demonstrated increased apoptotic cells by PCP4/PEP19 knockdown (n=3) 168 hr after siRNA transfection. *, p<0.05, **, p<0.01 and ***, p<0.001 versus with negative control siRNA. Si(−), negative control siRNA; Si(+), PCP4/PEP19 siRNA

Since PCP4/PEP19 is known as an anti-apoptotic peptide, we further investigated the decrease in viable cell counts upon siRNA treatment in MCF-7 and SK-BR-3 cells, to check whether they were due to an increase in apoptosis. Using the trypan blue exclusion test, we observed that the number of trypan blue-stained dead cells increased after PCP4/PEP19 knockdown, at 120 hr after siRNA transfection (Fig. 3A). By performing flow cytometric analysis, we found that sub G1 fractions of nuclear DNA content of cultured cells increased by PCP4/PEP19 knockdown in MCF-7 and SK-BR-3 cells, confirming that the decrease in viability count is due to increased occurrence of apoptosis (Fig. 3B). These dead cells exhibited DNA ladder formation in both MCF-7 and SK-BR-3 cells (Fig. 3C), indicating apoptotic DNA fragmentation. The increased numbers of cells harboring fragmented nuclear DNA in MCF-7 and SK-BR-3 cells after PCP4/PEP19 mRNA silencing was also confirmed by the TUNEL assay (Fig. 3D). All of these data indicated that PCP4/PEP19 knockdown increased apoptotic cell death in MCF-7 as well as SK-BR-3 cells.

### Effects of PCP4/PEP19 on CaMKK-Akt-Caspase and CaMKK/AMPK signaling

**Figure 4:**
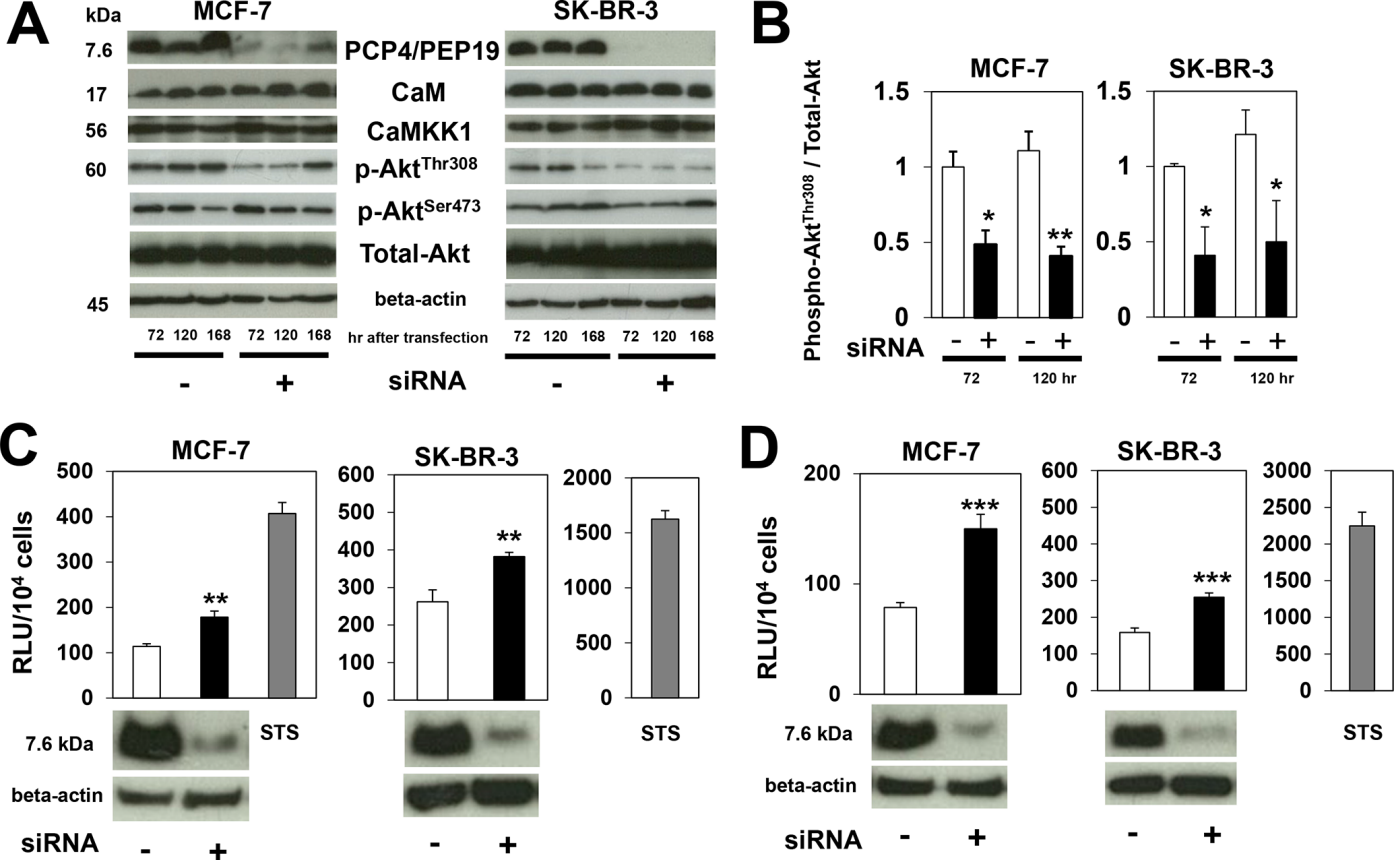
Expression of CaM, CaMKK1 and Akt, and caspase activities after PCP4/PEP19 knockdown **A**), **B**) Western blot analysis of molecules involved in CaM-CaMKK-Akt signaling pathway. After PCP4/PEP19 knockdown, expression levels of CaM, CaMKK1 and Akt did not change, but those of phospho-Akt^Thr308^ were significantly reduced in both MCF-7 and SK-BR-3 cells. The phospho-Akt^Ser473^ expression level was not decreased (n=3). **C**), **D**) In accordance with decreased phosphorylation of Akt^Thr308^, activities of caspase-3/7 (C) and caspase-9 (D) were increased 72 hr after transfection (n=6). STS treatment showed marked increase of caspase activities as positive control. *, p<0.05, **, p<0.01 and ***, p<0.001 versus negative control siRNA.

PCP4/PEP19 has been reported as a CaM-binding protein, through which PCP4/PEP19 could activate CaMKK signal cascades. Activated CaMKK1 then phosphorylates the Thr^308^ but not Ser^473^ residue of Akt, which in turn phosphorylates the Ser^136^ residue of Bad, finally leading to the suppression of caspase-3/7 and caspase-9 activation. After PCP4/PEP19 knockdown, levels of phosphorylated Akt^Thr308^ decreased, while those of Akt^Ser473^ remained the same (Figs. 4A and 4B). The total expression of CaM, CaMKK1, and Akt also remained as before. In accordance with the decreased phospho-Akt^Thr308^, caspase-3/7 and caspase-9 activities were significantly enhanced (Figs. 4C and 4D), which would be expected to result in a subsequent increase in the rate of apoptosis in both MCF-7 and SK-BR-3 cells, as was observed.

**Figure 5:**
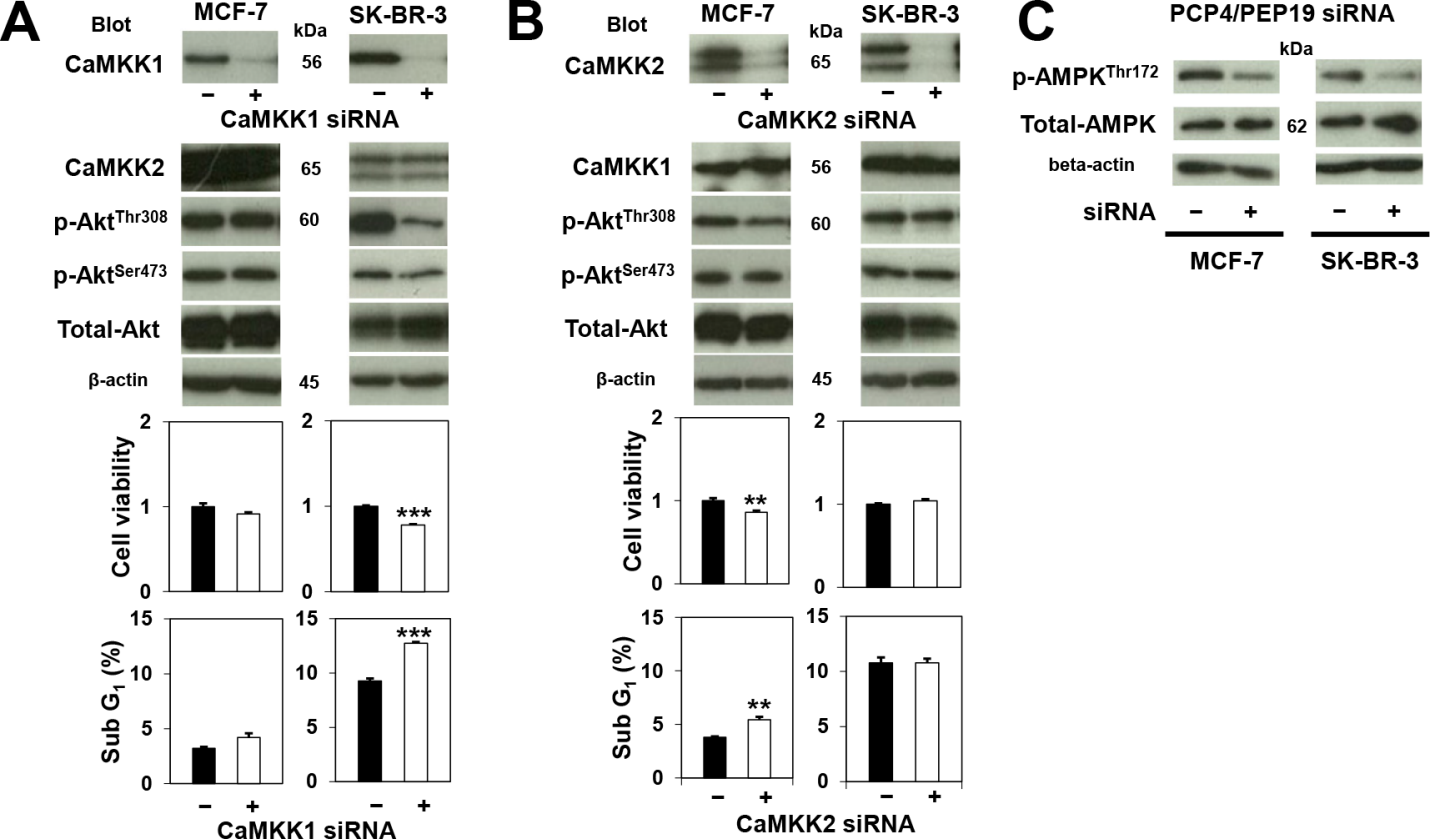
Effects of CaMKK knockdown on Akt and AMPK phosphorylation and cancer cell survival **A**) In MCF-7 cells, phosphorylation status of Akt^Thr308^, cell viability and apoptosis (sub G1 fraction) (n=3 to 6) were not affected by CaMKK1 knockdown. In contrast, CaMKK1 knockdown reduced the level of phospho-Akt^Thr308^ in SK-BR-3 cells, and apoptotic cell numbers were significantly decreased as a consequence. **B**) CaMKK2 knockdown, on the other hand, reduced Akt^Thr308^ phosphorylation in MCF-7, but did not reduce that in SK-BR-3 cells. The decreased phospho-Akt^Thr308^ corresponds to the occurrence of apoptosis (n=3). **C**) Phosphorylation of AMPK^Thr172^ was decreased only in SK-BR-3 cells by PCP4/PEP19 knockdown. **, p<0.01 and ***, p<0.001 versus negative control siRNA.

When CaMKK1 mRNA was silenced by siRNA in SK-BR-3 cells, Akt^Thr308^ phosphorylation and the numbers of viable cells significantly decreased, and those of sub G1 apoptotic cells increased (Fig. 5A, right panel). In contrast, CaMKK1 knockdown in MCF-7 cells showed no effect on Akt^Thr308^ phosphorylation, cell viability, or apoptosis (Fig. 5A, left panel). CaMKK2 knockdown in MCF-7 cells, however, showed a moderate decrease of Akt^Thr308^ phosphorylation, as well as a moderate decrease in cell viability due to apoptosis (Fig. 5B, left panel). In SK-BR-3 cells, no significant changes in cell viability, apoptosis frequency, or phosphorylation of Akt^Thr308^ were observed after CaMKK2 knockdown (Fig. 5B, right panel). The levels of AMPK^Thr172^ phosphorylation decreased by PCP4/PEP19 knockdown in SK-BR-3 cells, but not in MCF-7 cells (Fig. 5C).

### Expression and localization of PCP4/PEP19 in human breast cancer

**Figure 6:**
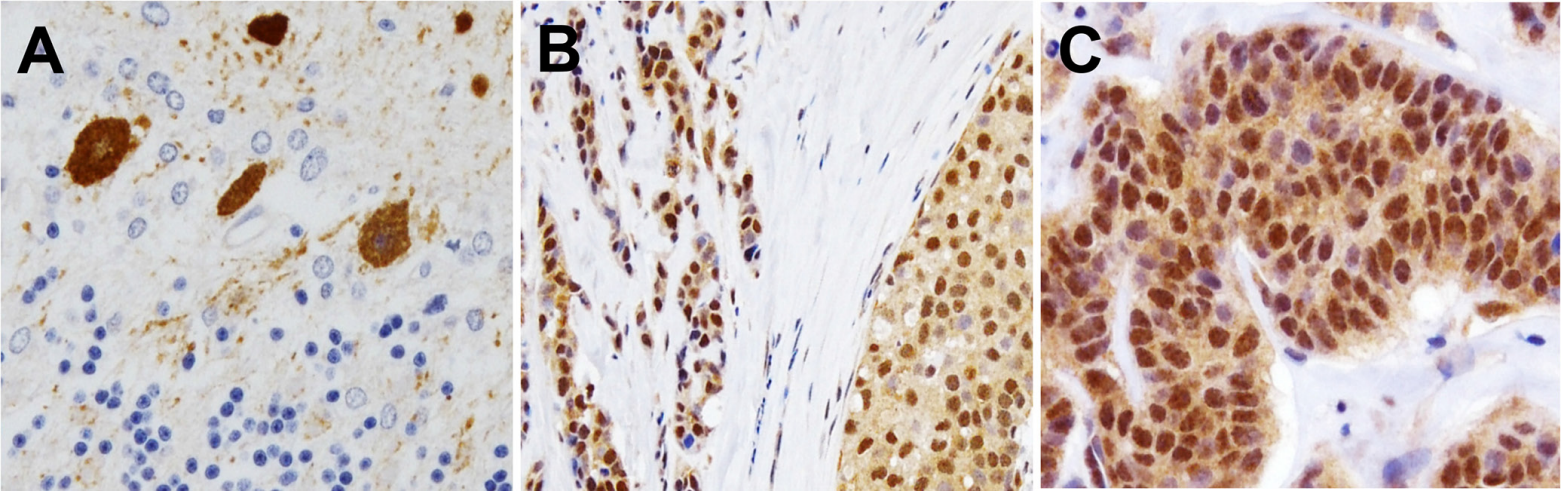
Expression and cellular localization of PCP4/PEP19 in human clinical cases of breast cancer tissues **A**) Human cerebellar Purkinje cells were specifically positive for the anti-PCP4/PEP19 antibody. **B**), **C**) In human cases of breast cancer tissues, PCP4/PEP19 was localized in both cytoplasm and nuclei of intra-ductal (B) and invasive parts (C).

Human cerebellar Purkinje cells were strongly positive for PCP4/PEP19 expression (Fig. 6A). Thirty cases of surgically resected invasive ductal carcinoma tissues were routinely processed by formalin-fixation and paraffin-embedding, and were used in immunohistochemistry assay to determine the expression and localization of PCP4/PEP19. Most of the cancer tissues (25/30, 83%) were positive for PCP4/PEP19 expression, which localized in both cytoplasm and nuclei. Both invasive and intraductal *in situ* components were positive for PCP4/PEP19 (Figs. 6B and 6C). The frequency of expression and cellular localization showed no significant correlation with the status of ER, progesterone receptor, or Her2 expression.

## DISCUSSION

Many strategies for cancer treatment, including breast cancers, are focused on new molecular targets, which regulate cell proliferation or cell death [18-20]. The specific aim of this study was to evaluate the expression and function of PCP4/PEP19 in relation to cellular proliferation of cultured human breast cancer cell lines. Herein, we demonstrated the anti-proliferative effects of PCP4/PEP19 knockdown mediated through decreased Akt phosphorylation and subsequent promotion of apoptotic cell death. Since the expression of PCP4/PEP19 was up-regulated by E2 stimulation in ER-positive MCF-7 cells, the cancer cell survival effects of E2 stimulation would be partially explained by the E2-induced expression of anti-apoptotic PCP4/PEP19 [21, 22]. SK-BR-3 cells that lack ER (therefore also lack E2 stimulated PCP4/PEP19 expression) constitutively and highly expressed PCP4/PEP19. Therefore, further characterization of these two human breast cancer cell lines can prove to be therapeutically significant, shedding light on E2-dependent and E2-independent mechanisms of cell survival that may underlie resistance to hormonal therapy. Furthermore, PCP4/PEP19 could potentially serve as a novel molecular target, serving to enhance apoptotic cell death irrespective of the status of ER expression, offering new treatment possibilities for breast cancers.

MDA-MB231 cells do not express PCP4/PEP19 but proliferate well, indicating that MDA-MB231 cells depend on other anti-apoptotic mechanisms to survive. By contrast, the dependence on PCP4/PEP19 of MCF-7 and SK-BR-3 cells could be explained by oncogene-addiction flood model [23-25]. Activation or over-activation of pro-survival pathway “A” (A corresponds to PCP4/PEP19 in MCF-7 and SK-BR-3 cells) may lead to deactivation of an alternative pro-survival pathway(s) because of redundancy. In these cells which are addicted to the pro-survival pathway “A”, targeting the pathway “A” will kill these cells.

In general, Ca^2+^-CaM-signaling is involved in many cellular events, such as cell proliferation, cell cycle transition, and cell survival through the inhibition of apoptosis (reviewed in ref. 26). Ca^2+^/CaM binding activates CaMKK1, which then phosphorylates Akt at the Thr^308^ residue, leading to Bad phosphorylation, caspase inactivation, and finally suppression of apoptosis [27]. The CaM signaling pathway and its function has been well studied in MCF-7 cells, in which CaM regulates Akt activity by its subcellular co-localization and inhibition of CaMK1 enhances oxidative stress-induced apoptosis [28, 29]. Inhibition of CaMKK1 and its substrate CaMK down streams of the CaM pathway result in G1 cell arrest [30]. PCP4/PEP19 is a CaM-binding polypeptide and acts as a modulator of CaM functions by regulating the association of Ca^2+^/CaM-dependent enzymes involved in the apoptosis signaling pathway in neural cells [9-11]. We showed that CaMKK1 knockdown resulted in decreased Akt^Thr308^ phosphorylation and a subsequent increase in apoptosis in SK-BR-3 cells. In MCF-7 cells; however, CaMKK1 knockdown neither reduced Akt^Thr308^ phosphorylation nor increased apoptosis, indicating that the CaMKK1/Akt pathway is not predominantly involved downstream of PCP4/PEP19 signaling and suggesting the possibility that PCP4/PEP19 is involved in other pathways that regulate apoptosis in MCF-7 cells.

CaMKK2 is known as an upstream activator of AMP-activated protein kinase (AMPK) and regulates cell metabolism through maintenance of energy balance, adiposity and glucose homeostasis [31, 32]. Some studies have reported CaMKK2 involvement in the pathophysiology of prostatic cancer, where CaMKK2 is identified as an androgen target gene and increased expression and activation of CaMKK2/AMPK signaling affects cell migration, invasion and anabolic pathways [33-35]. These findings indicate that the CaMKK2 signaling pathway may also be associated with prostatic cancer cell survival, in manner similar to that of the CaMKK1/Akt pathway, which has been suggested to play a role in cell survival in LNCaP prostatic cancer cells [36]. In human breast cancer SK-BR-3 cells, however, our results showed that CaMKK2 knockdown had no effects on apoptosis. Therefore, the CaMKK2/AMPK signal might not be involved in the cell survival mechanisms in human breast cancer SK-BR-3 cells, even though PCP4/PEP19 knockdown reduced AMPK phosphorylation. It has also been reported that activated-AMPK could have anti-apoptotic effects in MCF-7 cells [37, 38]. In MCF-7 cells, CaMKK2 knockdown actually enhanced apoptosis with moderate decrease of phospho-Akt^Thr308^, while PCP4/PEP19 knockdown did not decrease the phosphorylation of AMPK^Thr172^. Together, these results suggest that while PCP4/PEP19 is not necessarily an upstream regulator of CaMKK2/AMPK signaling, PCP4/PEP19, CaMKK2 and Akt are peripherally linked in a regulatory network, remaining to be fully elucidated, that modulates apoptosis in MCF-7 cells. Nevertheless, the anti-apoptotic role of PCP4/PEP19 possibly mediated through Akt-dependent mechanism is crucial to understanding the functions of PCP4/PEP19 in a wider context.

While the full extent of PCP4/PEP19 involvement in inhibiting the apoptosis signaling pathway in breast cancer cells is still to be elucidated, we hypothesized that PCP4/PEP19 expression in ER-negative SK-BR-3 cells may be a contributor to estrogen hypersensitivity that results from estrogen-deprivation resistance, through cross-talk with multiple signaling pathways, such as the mitogen-activated protein kinase (MAPK) and Akt [39-41]. This is particularly true in ER-dependent MCF-7 cells under a long-term estrogen-depletion culture. In these cells, the ER gets hyper-phosphorylated through the Akt pathway, compensating for estrogen depletion and resulting in enhanced expression of ER-target genes [42]. PCP4/PEP19 was constitutively and highly expressed in ER-negative SK-BR-3 cells, mimicking the ER-signal independent condition with a compensatory expression of anti-apoptotic PCP4/PEP19 for cell survival. Further study is necessary to clarify the biological role of PCP4/PEP19 using MCF-7 cells before and after the acquisition of estrogen-deprivation resistance and ER-signal independence.

PCP4/PEP19 was localized in both nuclear and cytoplasmic fractions in cancer tissues. This suggests that PCP4/PEP19 may play a dual role in cancer cells, each mediated through the cytoplasm and the nucleus separately. Supporting this, PCP4/PEP19 overexpression has been found to increase the transcription of a *CYP11B2* reporter gene in adrenocortical carcinoma cells that produce aldosterone [14]. The mechanism of PCP4/PEP19 nuclear translocation and its consequence remained to be clarified and are considered as future work. Extrapolating from the results obtained from the *in vitro* studies, it is probable that PCP4/PEP19 is highly expressed in human breast cancer tissues in clinical samples. Most of the tumor tissues were positive for PCP4/PEP19 irrespective of hormone receptor status, indicating that PCP4/PEP19 may present an attractive and alternative therapeutic target in breast cancer, especially in hormone-independent breast cancers.

In conclusion, our results indicate an anti-apoptotic function of PCP4/PEP19 in human breast cancer cells. These results, therefore, pose PCP4/PEP19 as a potential novel molecular target that may be used to suppress tumor cell growth in breast cancers via enhanced apoptosis, irrespective of the status of ER expression.

## METHODS

### Cells and cell culture

Human breast cancer MCF-7 cells were obtained from RIKEN BioResource Center (Tsukuba, Japan). SK-BR-3 and MDA-MB-231 cells were purchased from American Type Culture Collection (ATCC, Rockville, MD). The MCF-7, SK-BR-3 and MDA-MB-231 cells were grown in minimal essential medium (MEM, Sigma, St. Louis, MO), McCoy's 5A (Thermo Scientific, Waltham, MA) and Leibovitz's L-15 medium (ATCC) supplemented with 10% fetal bovine serum (FBS), respectively. MCF-7 and SK-BR-3 cells were maintained at 37°C with 95% air and 5% CO_2_. MDA-MB-231 cells were maintained at 37°C 100% air without CO_2_.

For removal of steroid hormones from FBS, a 5% charcoal and 0.5% dextran (Sigma) suspension in FBS was incubated at 37°C for 1 hr with shaking. The suspension mixture was then centrifuged at 2,500 rpm for 20 min, and the supernatant was filtered through a 0.2 μm filter.

### Antibodies and reagents

Anti-PCP4/PEP19 polyclonal rabbit antibody was obtained from Sigma. Antibodies against total Akt, phospho-Akt^Ser473^, phospho-Akt^Thr308^, total AMP-activated protein kinase (AMPK), phospho-AMPK^Thr172^, and beta-actin were from Cell Signaling Technology (Danvers, MA). Anti-CaM antibody was from Merck Millipore (Billerica, MA). Anti-CaMKK1 antibody was obtained from Novus Biologicals (Littleton, CO) and anti-CaMKK2 antibody was obtained from Abnova (Taipei, Taiwan). FITC-labeled anti-rabbit IgG antibody was used for immunofluorescence study (Cell Signaling Technology). Lipofectamine RNAiMax, a reagent for siRNA transfection was obtained from Life Technologies (Carlsbad, CA). 17-beta Estradiol (E2) and staurosporine (STS) were purchased from Sigma.

### Knockdown experiments by siRNA

Pre-designed siRNAs were used for knockdown of PCP4/PEP19 (ID: HSS181928), CaMKK1 (siRNA IDs: HSS130810, HSS130811, HSS130812), and CaMKK2 (IDs: HSS116423, HSS173805, HSS173806) mRNA, and Stealth RNAi™ siRNA Negative Control was used as negative control (Stealth RNAi™, Life Technologies). Cells were cultured in the growth media, and on the next day of plating, siRNAs (PCP4/PEP19: 25 nM, CaMKK1 and CaMKK2: 100 nM) were transfected by Lipofectamine^®^ RNAiMAX in Opti-MEM^®^ I (Life Technologies) with phenol red-free media containing 10% charcoal stripped FBS according to the manufacturer's instructions. On the next day of transfection, media were changed to phenol red-free media containing 10% charcoal stripped FBS, with or without E2.

### WST-8 assay

Cells were seeded at 10^4^ cells/well in 96-well plates with medium supplemented with 10% FBS, and allowed to attach overnight. On the next day after seeding, the media were replaced to phenol red-free media supplemented with charcoal stripped FBS. After E2 stimulation and/or siRNA transfection, Cell Counting Kit-8 (WST-8, Dojindo, Kumamoto, Japan) was used for viable cell counting according to the manufacturer's instructions at the indicated time. Cell viability was determined by measuring a generated formazan dye at 450 nm against a reference at 620 nm using a microplate reader.

### Trypan blue exclusion assay

Floating and attached cells were harvested, and then resuspended in medium. The cell suspension were mixed with 0.5% trypan blue stain solution. Cell numbers and viability were examined using a cell counter.

### Western blot analysis

Cells were washed with PBS and were precipitated with 10% trichloroacetic acid on ice for 30 min. Precipitates were washed with cold PBS and dissolved in cold lysis buffer (50 mM Tris-HCl (pH 6.8), 2% SDS, 10% glycerol, 6% 2-mercaptoethanol and 0.01% bromophenol blue). For detection of PCP4/PEP19, the lysates were analyzed using tris/tricine gels, and those of other proteins were completed using tris/glycine gels. After transfer onto polyvinylidene difluoride membranes, the membranes were blocked with 5% non-fat milk in TBST (20 mmol/L Tris-HCl (pH 7.6), 150 mmol/L sodium chloride, and 0.1% Tween 20) for 1 hr at room temperature (RT) and then incubated overnight at 4°C with primary antibody diluted in Can Get Signal solution 1 (Toyobo, Osaka, Japan) and with a horseradish peroxidase conjugated goat anti-rabbit antibody or goat anti-mouse antibody (MP Biomedicals, Santa Ana, CA) for 1 hr at RT. Protein expression was detected with SuperSignal West Pico chemiluminescent substrate or SuperSignal West Femto Maximum Sensitivity Substrate (Thermo Scientific). Densitometry analysis was performed with NIH ImageJ software version 1.43.

### Quantitative RT-PCR analysis of PCP4/PEP19

Total RNA was extracted using Total RNA Extraction Miniprep System (Viogene BioTek, New Taipei City, Taiwan) and stored at -80°C until analysis. Total RNA was converted into cDNA using a High Capacity RNA-to-cDNA Kit (Life Technologies). The cDNA was analyzed by a LightCycler^®^ 480 (Roche Diagnostics, Tokyo, Japan) and subjected to 45 cycles of amplification using TaqMan gene expression assays (Life Technologies). Each sample was analyzed in triplicate in separate wells for the target and reference *GAPDH* genes. The average of three threshold cycle values for the target gene and reference were calculated, then analyzed using the comparative Ct method. Custom made primers and TaqMan probe for PCP4/PEP19 gene amplification were purchased from Life Technologies (Assay ID: Hs01113638_m1).

### Flow cytometric analysis for cell cycle and apoptosis

Cells were trypsinized, and fixed in 70% ethanol at -20°C, then washed with phosphate-buffered saline. The cells were then incubated with phosphate-citrate buffer (pH 7.8) at RT for 30 min and resuspended in 1 mL of propidium iodide solution (50 μg/mL) containing 50 μL of RNase A solution (10 mg/mL). After suspensions were incubated for 30 min on ice, DNA content was analyzed using a CyAn-ADP flow cytometer (Beckman Coulter, Brea, CA).

### Terminal deoxynucleotidyl transferase biotin-dUTP nick end labeling (TUNEL) assay

The DeadEnd™ Fluorometric TUNEL system (Promega, Madison, WI) was used to detect apoptosis according to the manufacturer's protocol. These samples were subsequently stained for nuclei with propidium iodide (500 ng/mL) and were immediately analyzed using a CyAn ADP flow cytometer.

### DNA fragmentation assay

Genomic DNA was isolated by ApopLadder EX^®^ (Takara Bio, Shiga, Japan) and the fragmented DNA was amplified by ligation-mediated PCR method using DNA Ladder Assay PCR Kit (Maxim Biotech, Rockville, MD). The PCR products were analyzed by electrophoresis on a 1.5% agarose gel with detection by ethidium bromide staining.

### Caspase activity assay

Caspase-3/7, and caspase-9 protease activity was determined using Caspase-Glo 3/7 or 9 Assay Kit (Promega). Briefly, the cells, harvested by trypsinization, were washed and resuspened in PBS. Caspase-Glo 3/7 and Caspase-Glo 9 reagent containing luciferase and luminogenic substrates, Z-DEVD and Z-LEHD, respectively, were added, respectively. Then the reaction mixtures were incubated at 37°C for 30 min, and the intensity of luminescence was measured using a luminometer. Cells were treated with 1 μM STS for 3 or 6 hr for positive control of apoptosis and increased caspase activities.

### Immunohistochemistry

Immunohistochemistry for human breast cancer tissues resected at Hakuaikai Sagara Hospital was performed using formalin-fixed and paraffin embedded tissue sections. The rabbit polyclonal antibody raised against human PCP4/PEP19 and Vectastain Elite ABC kit (Vector Laboratories, Burlingame, CA) were used for the detection of PCP4/PEP19. As a positive control for immunohistochemistry, formalin-fixed and paraffin embedded tissue sections from human cerebellum containing Purkinje cells were used.

### Statistics

All data are presented as mean ± SE. Statistical significance was determined by unpaired one-tailed Student's *t* test and p < 0.05 was considered statistically significant.
